# Parasites Sustain and Enhance RNA-Like Replicators through Spatial Self-Organisation

**DOI:** 10.1371/journal.pcbi.1004902

**Published:** 2016-04-27

**Authors:** Enrico Sandro Colizzi, Paulien Hogeweg

**Affiliations:** Theoretical Biology and Bioinformatics, Utrecht University, Utrecht, The Netherlands; University of Texas at Austin, UNITED STATES

## Abstract

In a prebiotic RNA world, parasitic behaviour may be favoured because template dependent replication happens *in trans*, thus being altruistic. Spatially extended systems are known to reduce harmful effects of parasites. Here we present a spatial system to show that evolution of replication is (indirectly) enhanced by strong parasites, and we characterise the phase transition that leads to this mode of evolution. Building on the insights of this analysis, we identify two scenarios, namely periodic disruptions and longer replication time-span, in which speciation occurs and an evolved parasite-like lineage enables the evolutionary increase of replication rates in replicators. Finally, we show that parasites co-evolving with replicators are selected to become weaker, i.e. worse templates for replication when the duration of replication is increased. We conclude that parasites may not be considered a problem for evolution in a prebiotic system, but a degree of freedom that can be exploited by evolution to enhance the evolvability of replicators, by means of emergent levels of selection.

## Author Summary

The RNA world is a stage of evolution that preceded cellular life. In this world, RNA molecules would both replicate other RNAs and behave as templates for replication. A known evolutionary problem of this world is that selection should favour parasitic templates that do not replicate others, because they would be replicated the most. A possible solution to this problem comes from spatial self-organisation: local accumulation of parasites lead to their own local extinction, which leaves empty space for replicators to invade. We show that the spatial organisation generated by interacting replicators and parasites sets the (spatial) conditions that enhance replicase activity when parasites are stronger. Moreover, we find that the co-evolution of replicators and parasites is severely constrained by the type of spatial patterns they form, and we explore this feedback between evolution and self-organisation. We conclude that spatial self-organisation may have played a prominent role in prebiotic evolution.

## Introduction

According to the RNA world hypothesis, RNA self-replication preceded the current DNA-RNA-protein replication mechanism [1]. RNA molecules can store information much like DNA as well as catalyse reactions [2–4], including self-replication [5, 6], and are capable of undergoing Darwinian evolution [7–9]. From a theoretical view point, one of the simplest evolutionary systems consists of ribozymes that perform template dependent polymerization, even though such ribozymes are not fully functional yet experimentally [10, 11].

Replication *in trans* requires a catalytic molecule to bind and copy a template, and is thus prone to exploitation by molecules that behave more often as templates than as catalysts. At the extreme end of the spectrum lie parasites: RNAs that never replicate others and may be better templates than replicators [12, 13]. A well-mixed pre-biotic soup is indeed evolutionarily unstable because selection for better templates progresses until replicators cannot sustain themselves. Clearly, the evolutionary instability of this system is aggravated by parasitism. Some form of higher-level organisation is therefore necessary for the persistence of self-replicating RNAs.

We focus on the emergent levels of selection introduced by spatial self-organisation (a viable alternative is explicit compartmentalisation, such as vesicles [14–16]). The problem of parasites is alleviated in spatially extended systems due to spatial pattern formation [16–21], because parasites automatically segregate from replicators due to limited diffusion, and their accumulation leads to local, but not global, extinction. Furthermore, spatial patterns can constrain the evolutionary dynamics of parasites and select for weaker ones [20, 22, 23].

Here we show that parasites may not be a “problem” for the evolution of RNA-like self-replication in spatially extended system, but are actually beneficial, in that they sustain and enhance replication through higher levels of selection. This effect was briefly mentioned in [18]. Here we characterise it in terms of how the strength of parasites affects the selection pressures generated by spatial patterns. Building on this, we identify a phase transition-like behaviour, where the selection regime changes abruptly and we analyse the selection pressures at the replicators’ levels. Then, we explore the co-evolutionary dynamics of replicators and parasites.

## Methods

With an RNA-world in mind, we model the dynamics of a population of replicators and parasites in an individual based, spatially extended, stochastic simulation system. Individuals are located on a two-dimensional square lattice with eight neighbours and wrapped boundaries (based on CASH, [24]). Each node of the lattice can be empty or occupied by one individual. Replicators form complexes with other replicators at rate *k*_*a*_, and with parasites at rate *k*_*a*_ ⋅ *β* in order to replicate them (i.e. the replicator behaves as replicase). Assuming that replicators behave as templates with rate set to one, *β* ≥ 1 represents the relative advantage a parasite experiences as a template over replicators. Complexes occupy always two adjacent nodes. Complex dissociation happens with constant rate *k*_diss_. Upon successful complex formation between two adjacent individuals, and in the presence of empty space in the neighbourhood, the template is copied with rate *ρ*. After replication the complex breaks and the molecules return to an unbound state. Assume *X*_*i*_ is a replicator attempting to form a complex either with a replicator *X*_*j*_ or with a parasite *P*, the reaction scheme reads:

$${\text{X}}_{\text{i}}\;+\;{\text{X}}_{\text{j}}\overset{k_{a_{i}}}{\underset{k_{diss}}{⇌}}{\text{C}}_{{\text{X}}_{\text{j}}{{}^{\circ}\text{X}}_{\text{i}}}\overset{\rho ,\theta }{\to }2{\text{X}}_{\text{j}}\;+\;{\text{X}}_{\text{i}}$$

$${\text{X}}_{\text{i}}\;+\text{ P}\overset{\beta \cdot k_{a_{i}}}{\underset{k_{diss}}{⇌}}{\text{C}}_{\text{P}{}^{\circ}\text{X}}\overset{\rho \text{,}\theta }{\to }2\text{P }+\;{\text{X}}_{\text{i}}$$

where *C* is a complex and *θ* represents empty space. Mutations happen with probability *μ* and affect the complex formation rate *k*_*a*_ of the newly generated individual by adding a small random number (drawn from a uniform distribution $\left[-\frac{\delta_{\mu }}{2},\frac{\delta_{\mu }}{2}\right]$). Individuals degrade with rate *d*, leaving empty space (also when in complex, in which case the other molecule survives and returns to an unbound state). Diffusion is modelled by swapping node contents between neighbouring nodes, it happens with rate *D* and may involve single individuals as well as complexes. See S1 Text, Section S1 for more details.

Several important assumptions are made in order to simplify our model. First, we do not take into account that replication yields the complementary strand of a template. Second, we assume that replication rate *ρ* is the same for all replicators and parasites, and does not evolve in this study. Third, we assume that replicators behave as templates with rate set to one, which does not evolve, and parasites have are relatively more available for complex formation, i.e. *β* > 1. Notice that we let parasites’ relative advantage *β* evolve (where specified). Fourth, parasites are modelled as a different class of molecules, in line with the results of a previous study [25]. There, RNA-like replicators were modelled with sequences and secondary structures, and it was found that although few mutations could turn a replicator into a parasite, several mutations were necessary for parasites to optimise and establish themselves, thereby forming a new lineage. Furthermore, replicators evolved so that no close mutants of theirs was strongly parasitic [26]. Fifth, parasites do not form complexes with each other. In fact, complex formation was determined by sequence-complementarity in the above-mentioned study [25], and parasites evolved to minimise interactions with each other.

## Results

### 0.1 Individual-based selection decreases replication

**Fig 1:**
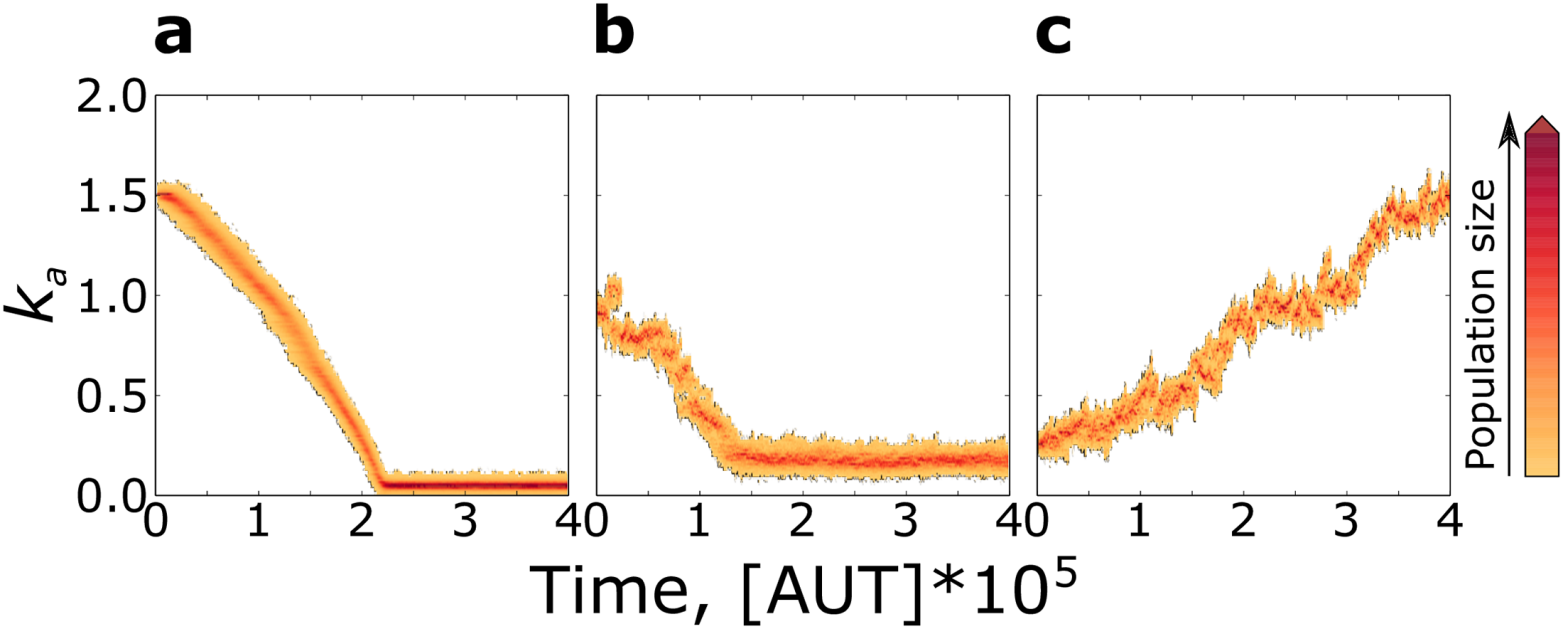
Evolutionary dynamics of replicators’ association rate without and with parasites: *k*_*a*_ decreases in the absence of parasites, and evolves to higher values the stronger the parasites. **a:** Only replicators are present in the system; **b:** Parasite advantage *β* = 1.40; **c:** Parasite advantage *β* = 1.70. Other parameters: *k*_*a*_ ∈ [0, 2], *k*_*diss*_ = 0.25, *ρ* = 1, Δ*t*_*r*_ = 0, *μ* = 0.005, *δ*_*μ*_ = 0.05, *d* = 0.03, *D* = 0.1. Results are robust to moderate changes in diffusion rate (S1 Text, Section S2.

When we let the association rate (*k*_*a*_) mutate in a replicators’ system without parasites, *k*_*a*_ decreases (Fig 1a) because replicators with lower-than-average *k*_*a*_ behave more as templates than as replicators, thus being replicated the most. Eventually, the minimum association rate needed for survival is reached (*k*_*a*_ ≈ 0.05, just above death rate, *d* = 0.03). There, the system persists indefinitely because mutations that further decrease the probability of complex formation lead to local extinction, followed by the invasion of neighbouring replicators. Mixing the system, as well as a large increase in the diffusion rate, leads to global extinction as *k*_*a*_ becomes too low to sustain replication in the system (S1 Text, Section S2).

### 0.2 A phase transition to high association rates for larger parasite strength

We now introduce parasites and let the evolutionary dynamics of the association rate *k*_*a*_ unfold in response to a large constant parasite advantage (*β* ∈ [1.0, 1.99]).

Importantly, parasites are at advantage over replicators because 1) complex formation automatically shifts replication towards parasites by introducing a replicase/template implicit trade-off for replicators [27], 2) they form complexes more frequently than replicators (*β* > 1) and 3) when in complex they do not replicate others, but are exclusively replicated. Yet, the deleterious effect of parasites is limited in spatial systems because local replication prevents them from becoming a global stability threat. The processes of spatial pattern formation in discrete replicators-parasites systems often results in travelling waves [16, 23], where replicators expanding into empty space constitute the wave-front, and parasites outcompeting those replicators make up the back. Parasites leave empty space behind themselves due to local extinction. Notice that, because *β* is a multiplicative term, larger *k*_*a*_ and larger *β* contribute synergistically to parasite replication. One could expect that lower rates of complex formation are selected in the face of stronger parasites until the system collapses and goes extinct.

**Fig 2:**
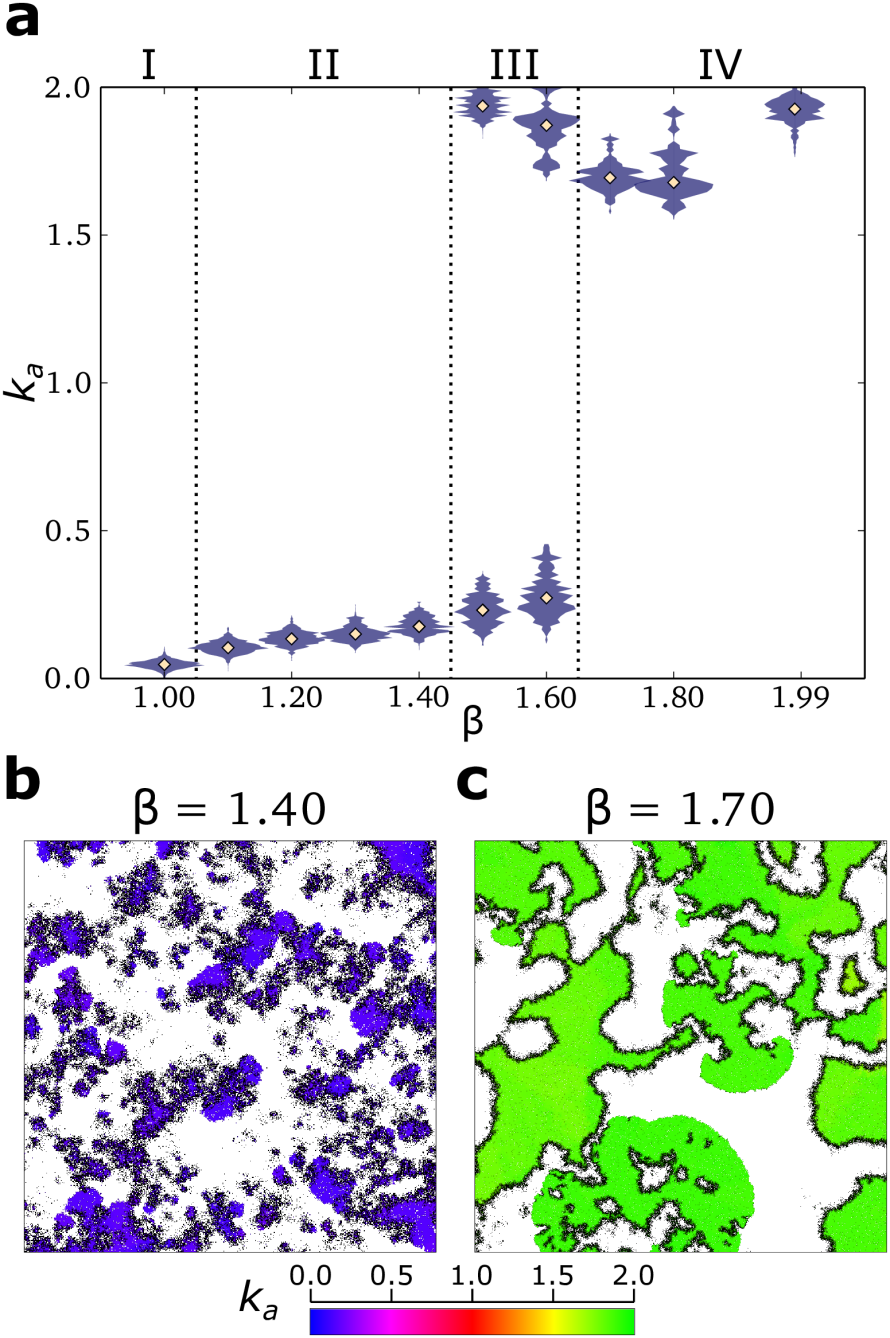
Stronger parasites lead to a phase transition in the eco-evolutionary dynamics. **a:** distribution of the association rate *k*_*a*_ at evolutionary steady state in a population of replicators, for different values of parasite advantage *β*. **b:** chaotic waves at evolutionary steady state when *β* = 1.40 (lattice size 512^2^, for clarity 1/4 of the lattice is displayed). **c:** stable waves at evolutionary steady state when *β* = 1.70 (lattice size 2048^2^, 1/4 of the lattice is displayed). All other parameters are as Fig 1.

Contrary to this expectation, replicators evolve to larger association rates in response to stronger parasites (Fig 1b and 1c). The increase in *k*_*a*_ is limited for lower parasite advantage (1.1 ≤ *β* ≤ 1.4), while for higher parasite advantage (*β* ≥ 1.7), *k*_*a*_ is maximised (Fig 2a). The system is bistable for intermediate values of *β* (1.5 ≤ *β* ≤ 1.6).

Parasites cannot be sustained in the system when they are too weak (*β* ≤ 1.0, S1 Text Section S3). For low values of both *β* and *k*_*a*_, the replication of parasites is comparable to that of replicators, while local accumulation of parasites leads to their extinction. After parasites disappear, *k*_*a*_ decreases to the minimum needed for survival (Fig 2aI).

**Fig 3:**
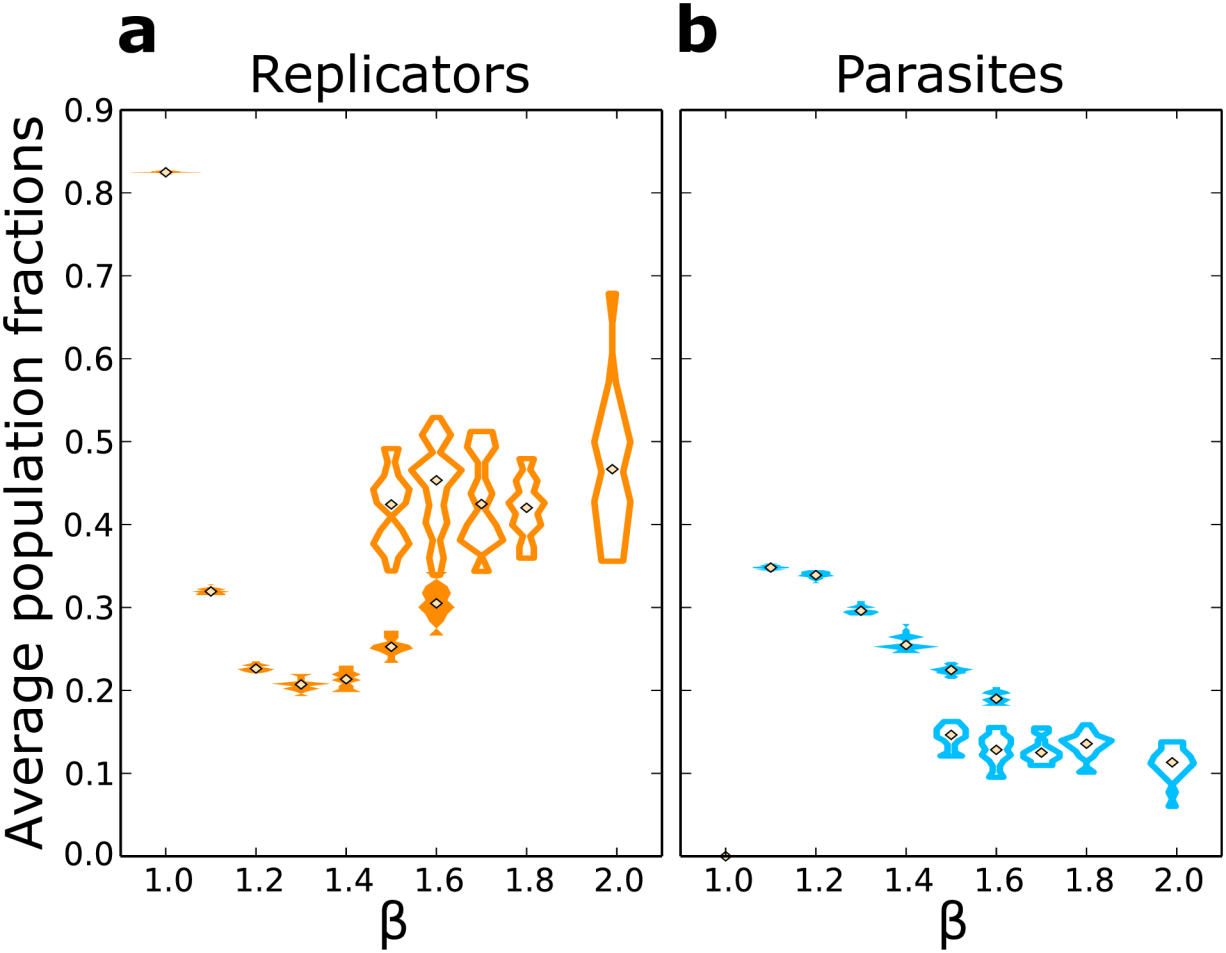
Fractions of a replicators and b parasites at evolutionary steady state. Full/empty distributions for replicators and parasites with the same *β* correspond in the two figures.

For increasing *β* (1.1 ≤ *β* ≤ 1.4, Fig 2aII), parasites persist and replicators reach an evolutionary steady state. Replicators and parasites organise in small and chaotic travelling waves, while the lattice looks overall patchy (Fig 2b). Observations of the spatial dynamics suggest that new waves are often established by replicators escaping from the back of an existing wave (Supplementary video S1 Video). Starting from large values of *k*_*a*_, replicators evolve to decrease it because those with smaller *k*_*a*_ generate new waves more frequently (S1 Text Section S4). *k*_*a*_ does not decrease without bound. Selection is stabilising because too low replication rates cannot support the persistence of both replicators and parasites. Surprisingly, the population size of replicators increases with parasite advantage (provided that *β* ≥ 1.3, Fig 3a). Notice that although parasites outnumber replicators at lower *β*, increasing parasite advantage steadily decreases the population of parasites (Fig 3b).

For 1.5 ≤ *β* ≤ 1.6 the system exhibits bi-stability, reaching either the lower or the higher (see below) final value of *k*_*a*_ depending on initial conditions (Fig 2aIII).

When parasite advantage is large, *β* ≥ 1.7, *k*_*a*_ is maximised over evolutionary time-scales (Fig 2aIV) and travelling waves become larger and stable (Fig 2c, Supplementary video S2 Video), provided that the lattice is large enough to contain them (S1 Text Section S5). As the value of *k*_*a*_ increases, parasites become stronger and it becomes progressively less likely that new waves establish by escaping from the back of an existing one. When waves with different replicators compete side by side, those where replicators have higher *k*_*a*_ win because they invade space faster than the others (S1 Text Section S6). Fig 3 shows that the population size of replicators is much larger than that of parasites, and that population sizes do not change much with increasing parasite strength.

In summary, weaker parasites lead to small and chaotic waves. Replicators evolve low association rates and establish new waves by escaping from the back of older waves (cf. [23]). In contrast, limited escape is possible from stronger parasites. Replicators respond by evolving higher association rates, and organise with parasites into larger and more stable travelling waves. The transition from one behaviour to the other is akin to a first order phase transition, even though the system is far from equilibrium.

### 0.3 Strong parasites induce high k_a_ by generating empty space

**Fig 4:**
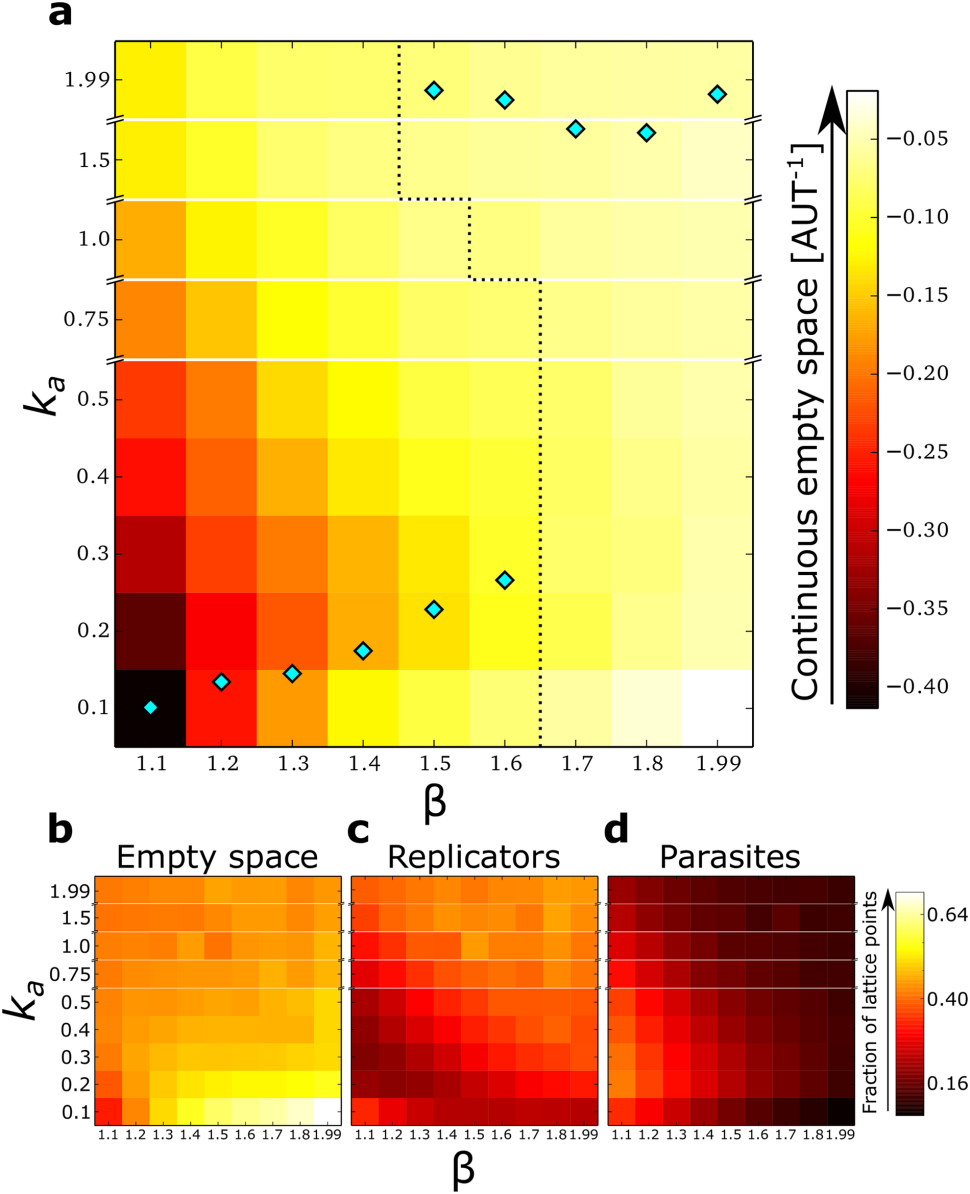
The amount of available empty space for waves predicts the phase transition in eco-evolutionary regimes better than other population statistics. **a:** Bifurcation diagram: Overlay of the median *k*_*a*_ (at the end point of the evolutionary dynamics) and the amount of uninterrupted empty space (from the ecological simulations). Cyan diamonds: median of the distributions of *k*_*a*_ after evolution, as taken from Fig 2; dotted line: tentative sketch of the separatrix between the two regimes; tiled *β* vs. *k*_*a*_ surface: colours are according to an index (see colour bar) that measures the amount of empty space in front of a wave, calculated from ecological simulations for a combination of *β* and *k*_*a*_ (see S1 Text Section S7 for details). **b:** Average fraction of lattice sites that are empty, **c:** occupied by replicators, **d** occupied by parasites, in the same ecological simulations used in **a**. All parameters as in Fig 1. For the ecological simulations *μ* = 0.

Since the two regimes differ in the way replicators and parasites organise in space (respectively chaotic vs. stable waves), we characterise the phase transition in terms of the amount of uninterrupted empty space experienced by the expanding fronts of travelling waves. To this end, we run several ecological experiment (i.e. without mutations) with different values of *k*_*a*_ and *β*, and (qualitatively) measure the amount of empty space experienced by replicators on the front of travelling waves (S1 Text Section S7). In Fig 4, the results from the ecological experiments and those from the evolutionary dynamics (Fig 2) are overlaid (the measure of the available empty space from the ecological system is represented as a heat-map for the different values of *k*_*a*_ and *β*).

For lower parasite advantage (*β* ≤ 1.60), the evolutionary outcome matches the values of *k*_*a*_ obtained from the ecological experiments where the amount of uninterrupted empty space is minimal (but not the total empty space, see Fig 4a), i.e. where wave birth is maximal. Thus, both wave-level selection (for higher wave birth) and replicator-level selection (for replication) lead to decreased *k*_*a*_ when it is large. When *k*_*a*_ is small, wave-level selection opposes individual-level selection, and *k*_*a*_ increases. A side effect of smaller steady state value of *k*_*a*_ is that replicators’ population size at evolutionary steady state is minimal (Fig 4b).

In contrast, for stronger parasites (*β* ≥ 1.70), replicators evolution maximises *k*_*a*_, and parasite population is smaller (Fig 4c). Fig 4 shows that uninterrupted empty space is abundant in this parameter region regardless of the values of *k*_*a*_. Because parasites are strong, replicators do not escape frequently from the back of the waves and do not form new waves, hence the large amount of uninterrupted empty space behind waves.

The availability of uninterrupted empty space drives the evolution of higher association rate, because sub-populations with higher *k*_*a*_ invade empty space faster and eventually dominate the expansion front.

**Fig 5:**
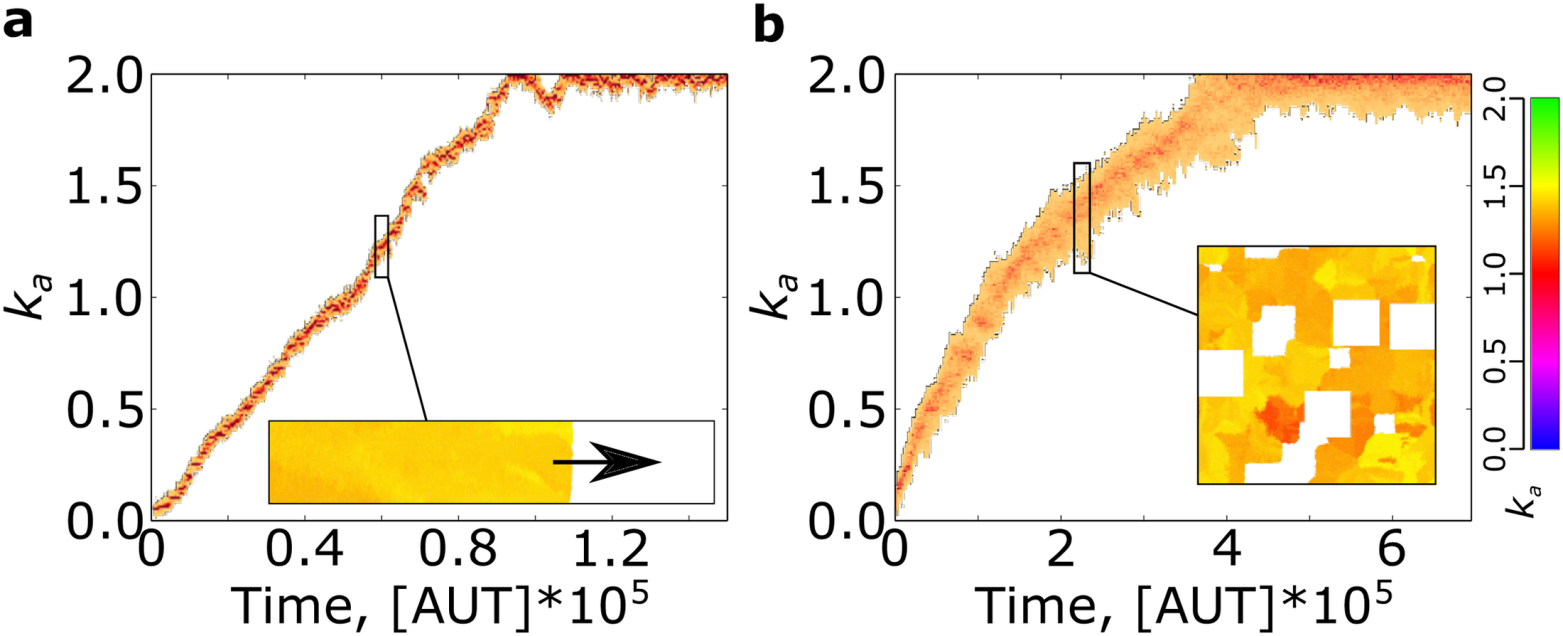
Association rate increases on the front of an expanding population, as well as when large scale disruptions occur. **a:** A population expands into an infinite space (Only the data about the front is plotted). Inset: a snapshot of the expansion dynamics (direction indicated by the arrow) **b:** evolutionary dynamics of *k*_*a*_ with large scale disruptions. Disruptions are as follows: every 50 AUT, 4 square patches (200^2^) of the lattice are turned to empty at random positions. Lattice size 1024^2^. Inset: a snapshot of the spatial dynamics.

We confirmed that *k*_*a*_ increases when empty space is available in two ways: by letting a population of replicators expand into unlimited empty space (Fig 5a), and by periodically disrupting a resident population of replicators with large scale ablations (Fig 5b, see S1 Text Section S8 for more details).

Altogether, we find that a positive feedback loop establishes: as populations of replicators evolve to higher *k*_*a*_ to colonise space faster, parasites benefit from increased complex formation, becoming stronger. As parasite strength increases, invadable space is increasingly perceived as limitless by replicators, which enables the further increase of *k*_*a*_. Thus, this process reverses the selection for becoming better templates.

In conclusion, strong parasitism leads to the formation of stable travelling waves, which in turn generate the selection pressure for increasing association rates in replicators.

### 0.4 Speciation as a consequence of spatial dynamics

So far we have shown that depending on the strength of the parasites, replicators evolve to moderate or high *k*_*a*_ through spatial self-organisation. Here we study replicator-only systems and show that in fact parasites arise automatically either as a consequence of disruptions or due to an increased cost of replication.

**Fig 6:**
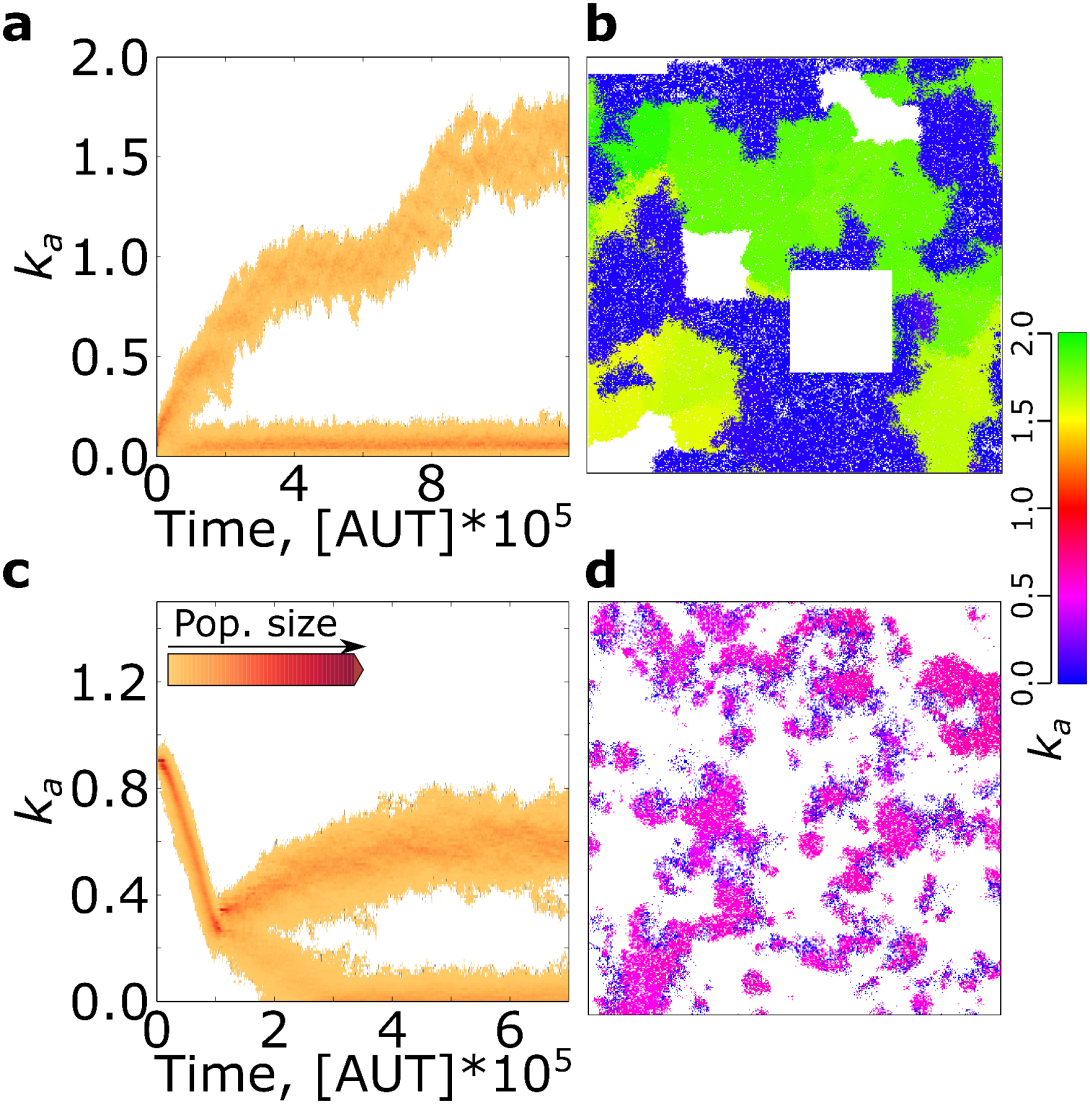
Smaller disruptions and increased cost of replication lead to speciation. **a:** Time plot of the distribution of *k*_*a*_ in a simulation with smaller disruptions. Disruptions are as follows: every 50 time steps, 16 square (100^2^) patches of the lattice are turned to empty at random positions. Lattice size 1024^2^, other parameters as in Fig 1. **b:** A snapshot of the simulation with disruptions (an area of 400^2^ is displayed). **c:** Time plot of the distribution of *k*_*a*_ in a simulation where duration of replication is Δ*t*_*repl*_ = 4 AUT. All other parameters are the same as above, except for *k*_*diss*_ = 0.1. **d:** A snapshot of the simulation with longer replication time (lattice size 1024^2^, displayed: 400^2^).

**Periodic disruptions can select for higher association rates and lead to the speciation of parasites.** Replicators evolve to large association rates with large scale disruptions (Fig 5b). In contrast, the evolutionary dynamics become richer due to speciation when a large number of patches with intermediate size are ablated: *k*_*a*_ increases in one lineage, while it decreases in the other, which behaves as a parasite (Fig 6a). Empty space allows for increasing *k*_*a*_ at the population’s expansion front, while selection for becoming parasitic occurs behind it (i.e. *k*_*a*_ decreases). Because the two selection pressures are spatially segregated, they can both be maintained and do not balance, hence speciation occurs. Parasite-like replicators survive when ablations are of intermediate size because they can expand to an area larger than the size of the ablation itself between two disruptive events (whereas they could not with larger ablations).

The persistence of the two lineages is dependent on the occurrence of disruptions, and a minimum ablation size exists for *k*_*a*_ to increase: microscopic perturbations, achieved by increasing decay rates, do not introduce sufficient continuous space for speciation to occur (S1 Text Section S8).

**Longer replication times induce speciation.** Besides externally imposed disruptions, larger scale heterogeneities can be autonomously generated in the system if replicators spend more time replicating another individual. The reaction scheme presented in Methods is modified so that ${\text{C}}_{{\text{X}}_{\text{j}}{}^{\circ}{\text{X}}_{\text{i}}}\overset{\rho ,\theta ,\Delta t_{repl}}{\to }{\text{2X}}_{\text{j}}\;+\;{\text{X}}_{\text{i}}$ and similarly for parasites.

So far, we have assumed that after a replicator and a template form a complex, replication is only limited by finding empty space before the complex breaks apart. With template-dependent polymerization in mind (e.g RNA replication), it is reasonable to assume that a complex spends time before replication is complete, e.g. undergoing conformational changes to activate the replication machinery, as well as spending time actually copying the template. Notice that longer replication times make replication more costly, because replicators spend a larger fraction of their life-time replicating others. This strengthens the selection pressure to decrease *k*_*a*_ (we assume that if a complex breaks before the replication time has passed, no product is formed).

When replication time is sufficiently long (Δ*t*_*repl*_ ≥ 3.5 AUT, S1 Text Section S9), a population of replicators undergoes speciation and two lineages form: in one *k*_*a*_ increases, in the other it decreases and these replicators behave as parasites (Fig 6c). The two species organise in travelling waves (Fig 6d) and establish an evolutionary feedback: parasite-like replicators cannot persist autonomously and exploit replicators with higher *k*_*a*_ for replication; the empty space they leave behind can be re-colonised by other replicators, which leads to increased *k*_*a*_.

Thus, longer replication times intensify the selection pressure to decrease *k*_*a*_. While in the previous paragraph empty space was imposed on the system, here large patches of empty space are generated when *k*_*a*_ becomes too low and replicators go extinct. The resulting invasion dynamics trigger the selection pressure for increasing *k*_*a*_ on the expanding front, which triggers the individual-based pressure to decrease *k*_*a*_ behind it and leads to parasite-like replicators. We conclude that the long term evolutionary consequence of longer replication time is that an emergent feedback establishes between evolution and spatial organisation. This feedback destabilises the evolutionary steady state presented in Fig 1a and two evolutionarily codependent species arise (cf. [21]).

Notice however that parasite-like replicators experience no relative advantage as templates (*β* is fixed to 1 in their case). The next question is, then, whether the co-evolution of replicators and parasites under a regime of more costly replication leads to stronger or weaker parasites, i.e. better or worse templates.

### 0.5 Longer replication time selects for weaker parasites in replicator-parasite co-evolution

We extend the replicator-parasite system presented above by letting the parasite advantage *β* co-evolve with the replicators’ association rate (*k*_*a*_), and we vary the duration of a replication event (Δ*t*_*repl*_).

**Fig 7:**
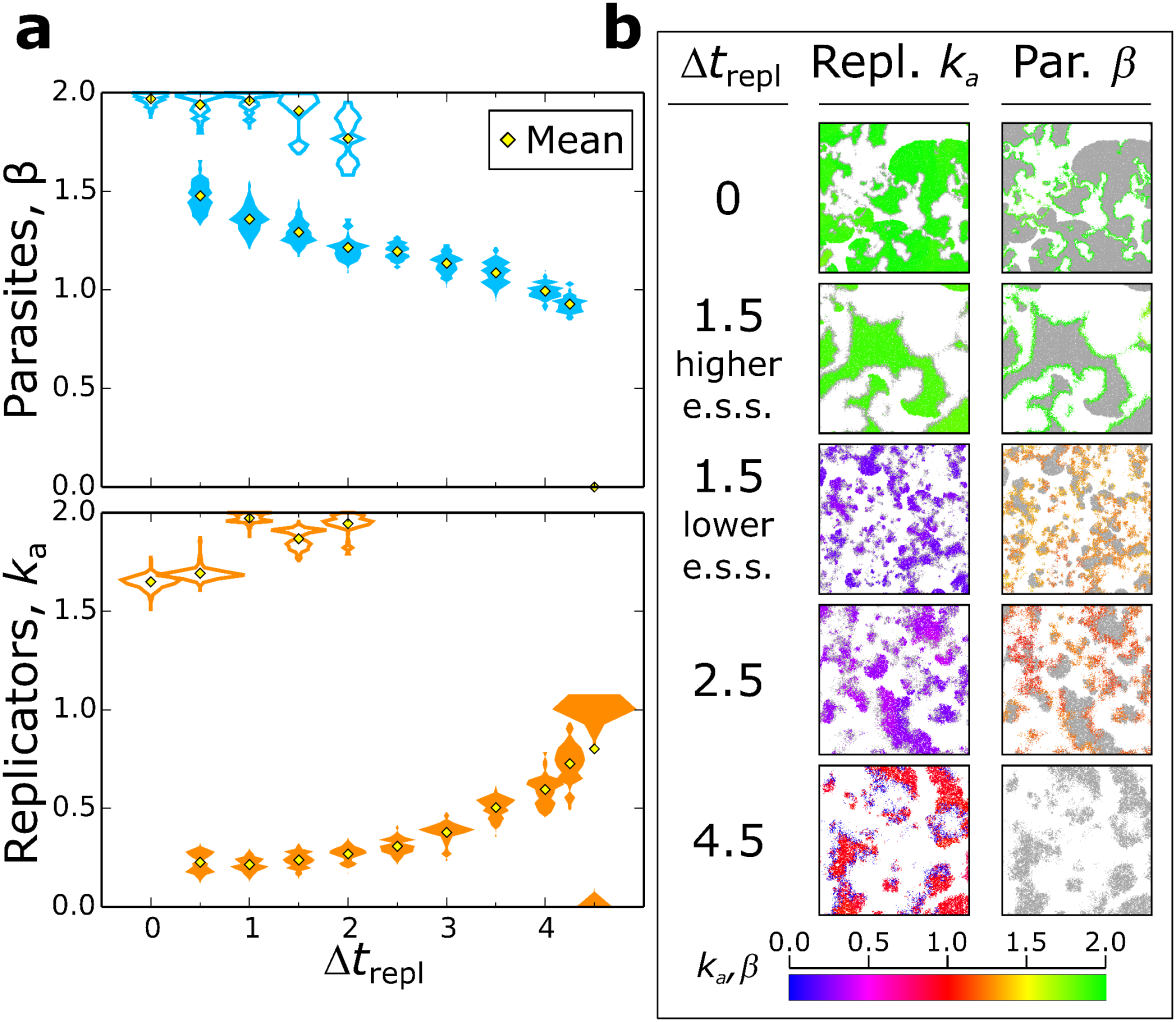
Bistability in the co-evolutionary steady states of replicators (*k*_*a*_) and parasites (*β*), in response to longer replication times. **a:** A full/empty distribution in the parasite pane corresponds to the full/empty distribution for replicators at the same Δ*t*_*repl*_. All parameters as above, *β* ∈ [0, 2]. **b:** Snapshots of the lattice from simulations with different Δ*t*_*repl*_. Left snapshot: spatial distribution of replicators (parasites in grey); right snapshot: parasites (replicators in grey). Lattice size from top to bottom: 2048^2^, 1024^2^,1024^2^,512^2^,512^2^ (1/4 of the lattice is displayed for clarity).

**Co-evolutionary dynamics.** In Fig 7 the co-evolutionary steady state values of *β* and *k*_*a*_ are plotted for different replication times.

**Fig 8:**
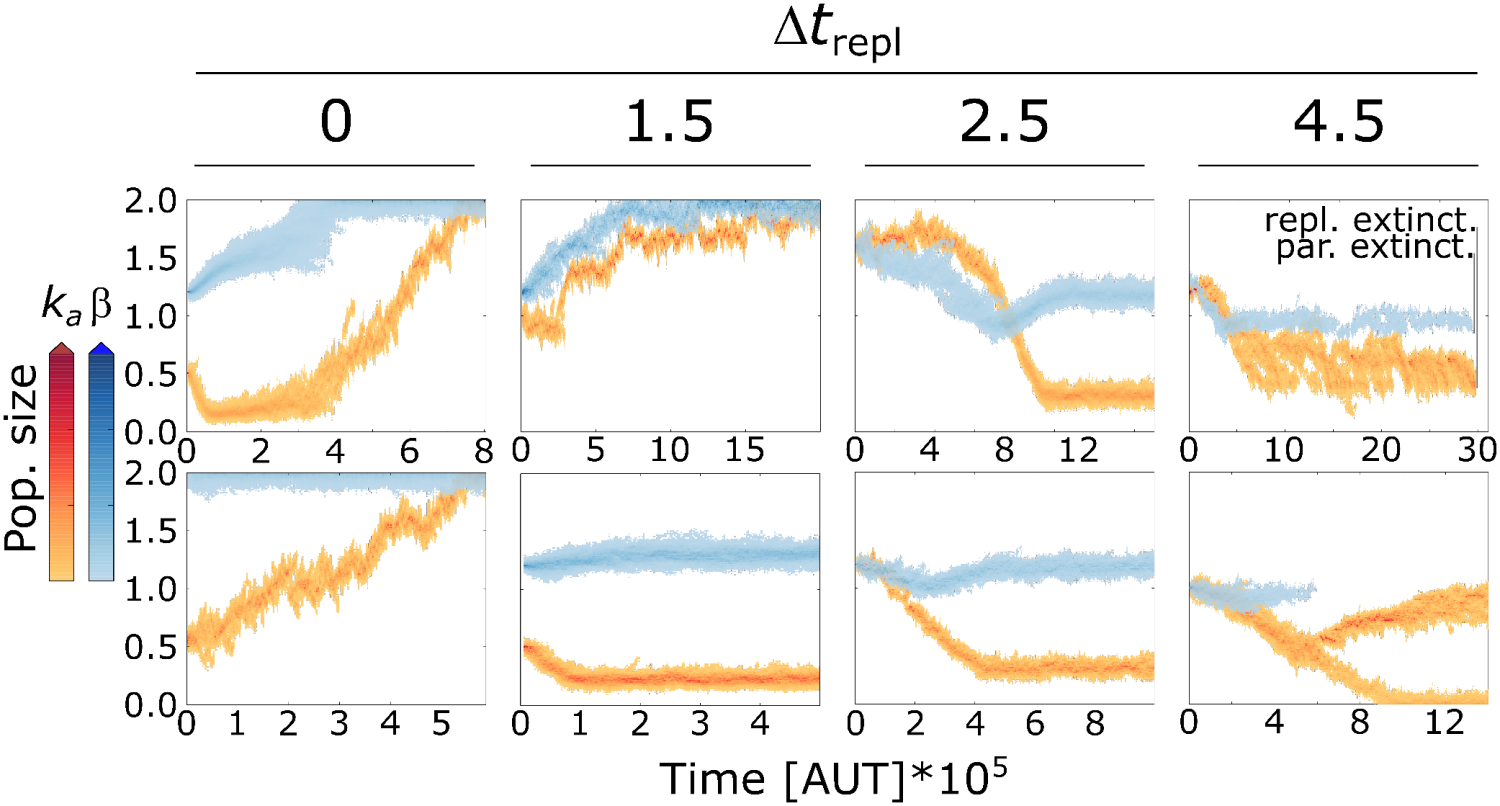
Bistability in the co-evolutionary steady states of replicators and parasites, in response to longer replication time. Pictures show the co-evolutionary trajectories of *β* and *k*_*a*_ for different values of Δ*t*_*repl*_ and different initial conditions.

The replication rates of both replicators and parasites is maximised in the long term co-evolutionary dynamics when replication time Δ*t*_*repl*_ is set to zero (Fig 7a), i.e. the setting used for the replicators-parasites system analysed above. While parasites are selected to constantly increase *β*, the evolutionary trajectory of *k*_*a*_ depends on initial conditions (Fig 8): when *k*_*a*_ and *β* are initialised at lower values, *k*_*a*_ first remains stationary around a (meta)stable equilibrium line (cf. Fig 4), and reaches larger values only when *β* is large enough. Taken together, the evolutionary trajectory of *k*_*a*_ and observation of the spatial dynamics show that the system autonomously (dynamically) undergoes the phase transition between chaotic and stable waves described in Fig 2.

The evolutionary maximisation of both *β* and *k*_*a*_ occurs for replication times up to Δ*t*_*repl*_ = 2.0.

However, a second steady state emerges for Δ*t*_*repl*_ > 0 for which *β* and *k*_*a*_ are not maximised, but rather they stabilise at lower values (Fig 7a). Following this steady state to larger Δ*t*_*repl*_, we observe that *β* decreases and *k*_*a*_ increases, i.e. weaker parasites and stronger replicators evolve. When Δ*t*_*repl*_ ≥ 4.0 the median value of *β* is less than one, i.e. parasites become worse templates than replicators. For Δ*t*_*repl*_ = 4.5 parasites evolve to extinction, and replicators may speciate into two lineages, one of which becomes parasite-like (*k*_*a*_ approaches zero, see previous paragraph).

*Spatial population dynamics.* The spatial co-evolutionary dynamics that lead to these steady states are straightforward.

For *β* and *k*_*a*_ sufficiently large, and lower Δ*t*_*repl*_, replicators and parasites organise into stable travelling waves (Fig 7b, Δ*t*_*repl*_ = 0 and the higher steady state of Δ*t*_*repl*_ = 1.5). There, parasites with larger *β* outcompete those with lower *β* because the former form more complexes with replicators, and therefore invade faster. The empty space generated behind parasites is then occupied faster by replicators with larger *k*_*a*_. Meanwhile, parasites with larger *β* profit the most from replicators with larger values of *k*_*a*_. This leads to maximise both replicators and parasites’ replication rates (Fig 8).

In contrast, for longer replication times (Fig 8, Δ*t*_*repl*_ > 1.5) or with smaller initial values of *k*_*a*_ relative to *β* (Fig 8 Δ*t*_*repl*_ = 2.5, lower pane), the spatial dynamics of this steady state are characterised by chaotic travelling waves. New waves are established by replicators creeping through the back of older waves (Fig 7b,Δ*t*_*repl*_ = 1.5-lower steady state, Δ*t*_*repl*_ = 2.5 and 4.5). Chaotic waves cause the co-evolutionary stabilisation of the replication rates of both parasites (cf. [23]) and replicators (see above). This effect is striking because a larger Δ*t*_*repl*_ leads to both a stronger selection pressure on replicators for decreasing *k*_*a*_, and a larger intrinsic advantage to parasites (because they do not pay any cost for replication). Yet, increasing Δ*t*_*repl*_ leads to co-evolutionary steady states where replicators’ association rate is larger and parasites’ advantage as templates is smaller.

*Spatial pattern formation causes directional changes in the evolutionary trajectories of replicators.* The selection pressure on replicators can change direction during the co-evolutionary dynamics, making the evolutionary trajectory of *k*_*a*_ non monotonic (Fig 8 upper row). This happens because spatial patterns themselves change over the course of evolution (cf. [28]).

Although the evolutionary steady state with low *k*_*a*_ is not stable for Δ*t*_*repl*_ = 0, its “ghost” (cf. [29]) can be observed in the trajectory of the co-evolutionary dynamics (Fig 8). It can take repeated “evolutionary attempts” for the system to transition completely to the phase with stable waves and replicators with higher *k*_*a*_ (S1 Text Section S10), because smaller waves locally outcompete larger ones (cf. [23]).

For Δ*t*_*repl*_ = 1.5 a similar effect can be seen, where replicators initially decrease their *k*_*a*_ in response to weak parasites, but then increase *k*_*a*_ when parasites have evolved to sufficiently high *β* (Fig 8). Interestingly, parasites are initialised at the same value in the two panes of Fig 8 for Δ*t*_*repl*_ = 1.5. Their evolution is dependent on the kinds of replicators they form waves with. Because in both cases replicators face “weak” parasites, they respond by decreasing *k*_*a*_. However, in one case replicators start with high *k*_*a*_ so parasites increase *β*, in the other replicators have low initial *k*_*a*_ and parasites do not maximise *β*.

The opposite situation may also happen for longer replication times (Fig 8, Δ*t*_*repl*_ > 1.5), replicators initially respond to strong parasites by increasing *k*_*a*_. Later, when *β* is sufficiently small, replicators are selected to decrease *k*_*a*_ (notice that although *β*’s trajectories are non monotonic, parasites evolve a monotonically decreasing complex formation rate *β* * *k*_*a*_). When replication time is long enough and the initial value of *k*_*a*_ is low relative to *β*, travelling waves destabilise because parasites invade replicators faster than replicators expand into empty space (the expansion front becomes narrower). In these limit conditions (if parasites were any stronger the system would go extinct) replicators cannot escape from the back of older waves and initially evolve to larger *k*_*a*_, while the parasitic erosion of the invasion front often isolates small groups of replicators from the rest of the wave (S1 Text Section S11), which survive longer if the associated parasites are weaker.

Finally, the limit viable replication time is Δ*t*_*repl*_ = 4.5, for which parasites drive themselves to extinction (global extinction quickly ensued for Δ*t*_*repl*_ = 4.7 and larger). When parasites are extinct, replicators cannot benefit from the selection pressure that sustained higher *k*_*a*_ (deriving from spatial pattern formation) and may themselves face extinction (Fig 8, Δ*t*_*repl*_ = 4.5 upper pane). If parasites are not allowed to evolve *β* below 1, instead, both replicators and parasites persist indefinitely (S1 Text Section S12). Replicators can persist in the absence of parasites if they succeed at evolving a parasite-like lineage and re-organise in spatial pattern (Fig 8, Δ*t*_*repl*_ = 4.5 lower pane).

In conclusion, the emergent selection pressures originating from self-organised spatial patterns locks the evolution of replicators to the evolution of parasites. This either leads to stable travelling waves that allow for the evolutionary increase in replication rates of both replicators and parasites, or to chaotic travelling waves that bring the system to an evolutionary pressure that selects for weaker parasites and stronger replicators, when the cost of replicating others becomes higher. We stress that results are due to the co-evolution of replicators and parasites, which allows a larger degree of complexity to unfold. In fact, these results are lost when only replicators can evolve at longer replication times, and instead we obtain results qualitatively similar to Fig 2, namely that stronger parasites lead to higher association rates in replicators (S1 Text Section S13).

## Discussion

In this study we analysed the eco-evolutionary dynamics of minimal replicator-parasite systems. Replicators copy templates after forming complexes with them, and parasites may be better templates than replicators.

An earlier study on a similar system [23] showed that when only parasites could mutate, selection for wave-level fertility (i.e. small and chaotic waves) resulted in the evolution of weaker parasites. Here we have paralleled that result when replicators mutate: replicators evolve to smaller association rates when parasites advantage is weak by escaping more frequently from the back of their waves, which forms more (smaller and chaotic) waves. Whether parasites or replicators mutate, waves evolve so that new waves are generated more easily. Hence there is selection for wave-fertility.

However, we have also identified a novel mode of evolution when parasites are stronger, which produces stable and long-lived travelling waves composed of replicators with larger association rates. Therefore, waves can also experience selection for longevity.

A phase transition separates these two spatial patterns and their evolutionary regimes. This phase transition can be studied by measuring the amount of empty space generated by parasites, and invaded by replicators at the front of a travelling wave (cf. [21, 30–33]).

We conclude that although replicators are prone to decrease association rates (to spend more time as templates), introducing parasites allows replicators to sustain a higher association rate.

Notice that 1) we recover both evolutionary regimes when we set *k*_*a*_ to constant and let only parasites mutate (S1 Text Section S14) and 2) longer replication time-spans Δ*t*_*repl*_ > 0 do not qualitatively change these results.

Finally, we analysed the co-evolution of replicators and parasites when the time-span needed for replication is prolonged. For smaller Δ*t*_*repl*_ both evolutionary strategies are attainable, and the system shows evolutionary bi-stability. For larger Δ*t*_*repl*_, only the co-evolutionary steady states with relatively lower *k*_*a*_ and *β* is reachable.

We introduced the term Δ*t*_*repl*_ to study the evolutionary dynamics of the system when replication rates do not depend solely on the availability of empty space. However, we did not let Δ*t*_*repl*_ evolve because we did not specify any molecular detail of RNA replication (for instance, the evolution of larger or smaller Δ*t*_*repl*_ should be functionally related to that of *β* and *k*_*a*_), which instead could be better targeted by sequence-based models [17, 25, 26].

Nevertheless, let us assume that Δ*t*_*repl*_ scaled with template length, i.e. Δ*t*_*repl*_ > 0, and that *k*_*a*_ and *β* evolved independently from it. Because Δ*t*_*repl*_ would likely be selected to decrease, following the lower equilibrium line of Fig 7, evolution would reach a steady state in which neither *k*_*a*_ nor *β* are maximised. Thus, our results would hold and we would recover the conclusions of [23].

### Pre-biotic evolution

In the context of prebiotic evolution, mutually replicating molecules (among which e.g. hypercycles [34]) are known to be evolutionarily unstable: selection at the individual level causes the evolution of better templates to the detriment of replication [12, 13]. Higher-order organisation, such as spatial extension ([17, 35]), spatial pattern formation ([16, 19, 36]) or vesicles ([14, 15]), is often invoked to solve this problem.

Here we have shown that individuals that behave only as templates may actually aid the evolution of higher replication rates. Parasitic behaviour is, in fact, “functional” because it contributes to the spatial structure that selects for higher levels of replication.

An earlier study on metabolism-based replicator models [20] showed that metabolic parasites could evolve into replicases, providing a group-level (albeit relatively costless) evolutionary benefit to the system. In our system instead parasites are beneficial *as parasites*, since we do not pre-conceive extra functional possibilities for them. Parasites induce more replicase activity in pre-existing replicases despite the cost of being a stronger replicase.

We conclude that parasites may be considered a functional degree of freedom that can be exploited by evolution through higher-order organisation.

### The evolution of multilevel evolution

In general, spatial pattern formation can deeply affect the evolution of its components [19, 37, 38], and can lead to selection that reinforces the spatial patterns even at the expenses of its composing individuals [39], or to self-organised evolutionary switching between different spatial patterns [28]. This higher-level selection can be recognised in travelling waves as well [16, 18, 23]. Travelling waves, however, also display compositional (and spatial) inheritance and variation, and therefore are units of evolution ([23]).

Here we have shown that the system can autonomously undergo the phase transition between chaotic and stable waves, as a result of a feedback between evolution and self-organisation. This means that the self-reinforcing selection pressure can change directionality. Thus we have observed the evolution of wave-level evolution, transitioning from selection for fertility to selection for longevity.

It has been recently asked: “How far can the RNA World go without being encapsulated in a cell?” [40]. We have not yet seen its limit, it seems.

## Supporting Information

S1 Text Supplementary Material. Additional data and results.
(PDF)

S1 Video Chaotic travelling waves. The video shows the dynamics of the system at evolutionary steady state for *k*_*a*_, when *β* = 1.40. In this regime the system organises into chaotic travelling waves.
(MP4)

S2 Video Stable travelling waves. The video shows the dynamics of the system at evolutionary steady state for *k*_*a*_, when *β* = 1.70. In this regime the system organises into stable travelling waves.
(MP4)

S1 File Source code. The source code used to run the simulations.
(ZIP)
