# Supplementary material for: Parasites Sustain and Enhance RNA-Like Replicators through Spatial Self-Organisation
Source: PLoS Comput Biol. 2016 Apr 27;12(4):e1004902. doi: 10.1371/journal.pcbi.1004902 (PMC4847872; doi:10.1371/journal.pcbi.1004902)
Supplement: S1 Text — Additional data and results. (PDF) [file pcbi.1004902.s001.pdf]

Supplementary material of Parasites Sustain and Enhance RNA-like Replicators Through Spatial Self-Organisation

Enrico Sandro Colizzi[1,2], and Paulien Hogeweg[1]

April 7, 2016

1 Affiliation: Theoretical Biology, Utrecht University, Utrecht, The Netherlands

2 Corresponding author: e.s.colizzi@uu.nl

S1 Details of the model

The model is a spatially extended, individual oriented Monte Carlo simulation system. Space is modelled as a two-dimensional square lattice with toroidal boundaries (based on CASH, de Boer and Staritsky (2000)). Each node has eight neighbours (the Moore neighbourhood) and can be empty or occupied by one individual. All dynamics implemented is local, involving only nodes in the Moore neighbourhood, and all nodes are updated in random order in one iteration of algorithm.

Replicators and parasites' complex formation and replication There can be two types of individuals, replicators and parasites. They differ in two ways: 1) replicators can both *form* and *accept* complexes, whereas parasites can only *accept* complexes; it follows that replicators can form complex with each other and with parasites, but the latter can never form complexes with other parasites. 2) Parasites can be better than replicators at forming complexes. This is modelled by assuming that replicators accept complexes with rate scaled to 1, while parasites accept complexes at rate $\beta \geq 1$ (unless it evolves otherwise), which represents the advantage parasites experience at accepting complexes relative to replicators. The rate at which replicators form complexes, called the "association rate" throughout the text, is k_a. Complex molecules always occupy two adjacent nodes of the lattice (and each molecule in complex keeps a "flag" that signals where the other molecule is located).

Complex dissociation happens with constant rate k_{diss}. When a complex is formed between two adjacent individuals, Δt_{repl} AUT (units of time) must pass without the complex dissociating; then if an empty node is available in the neighbourhood, the template is copied with rate ρ. After replication, the complex breaks and molecules return to an unbound state. Notice that the time passing, i.e. the counter that starts from Δt_{repl} and reaches zero, is modelled as a first order reaction. Individuals decay with rate *d*, leaving empty space. Molecules in complexes can also degrade.

Assume X_i and X_j are replicators, P is a parasite, C is a complex (the suffixes indicate the individuals in complex, as well as which one is template and which one is replicase) and θ represents an empty node. The full reaction scheme reads:

$$\mathrm{X_i} + \mathrm{X_j} \begin{cases} \underset{k_{diss}}{\overset{k_{a_i}}{\rightleftharpoons}} \mathrm{C_{X_j \circ X_i}} \xrightarrow{\rho,\theta,\Delta t_{repl}} 2\,\mathrm{X_j} + \mathrm{X_i} \\ \underset{k_{diss}}{\overset{k_{a_j}}{\rightleftharpoons}} \mathrm{C_{X_i \circ X_j}} \xrightarrow{\rho,\theta,\Delta t_{repl}} 2\,\mathrm{X_i} + \mathrm{X_j} \end{cases}$$

$$\mathrm{X_i} + \mathrm{P} \underset{k_{diss}}{\overset{\beta\cdot k_{a_i}}{\rightleftharpoons}} \mathrm{C_{P\circ X}} \xrightarrow{\rho,\theta,\Delta t_{repl}} 2\,\mathrm{P} + \mathrm{X_i}$$

$$\mathrm{X,P} \xrightarrow{d} \theta$$

$$\mathrm{C_{X\circ X}} \xrightarrow{2d} \mathrm{X}$$

$$\mathrm{C_{P\circ X}} \xrightarrow{d} \mathrm{X}$$

$$\mathrm{C_{P\circ X}} \xrightarrow{d} \mathrm{P}$$

Mutations happen after replication with probability μ and may affect the association rate k_a of the newly generated replicator, or parasite advantage β, by adding a small random number drawn from a uniform distribution $[-\frac{\delta_\mu}{2}, \frac{\delta_\mu}{2}]$. No other parameter is evolved.

Diffusion Diffusion is modelled by swapping the contents of neighbouring nodes, it happens with rate D and may involve single individuals as well as complexes. The algorithm for diffusion follows Takeuchi and Hogeweg (2009): diffusion between two molecules or a molecule and empty space is modelled as a second order reaction, diffusion between a complex (which occupies two lattice nodes) and a free molecule or an empty node is considered a third order reaction,

diffusion between two complexes is a fourth order reaction. Suppose that the focal molecule X_1 is in complex with X_2, and a randomly chosen neighbour Y is a single molecule. Diffusion swaps their locations in order to maintain adjacency between molecules in complex as follows: X_1 takes the place of Y, X_2 takes the place where X_1 was, and Y is moved to the node where X_2 was. Two complexes, $X_1 \circ X_2$ and $Y_1 \circ Y_2$, swap places as follows: assuming X_1 is the focal molecule, and Y_1 the random neighbour, X_1 moves to the node of Y_2 and vice versa, X_2 moves to Y_1's location and vice versa.

Algorithm update One iteration of the alogrithm runs as follows.

- One node is drawn randomly, and one if its neighbouring nodes is selected randomly.
- Based on the content of the nodes, the possible events are determined, e.g. a replicator next to a parasite can form a complex, swap places or decay.
- One event is drawn. Each event happens with probability proportional to its rate, scaled by a constant which is larger than the sum of all the rates of all possible reactions (because one event is drawn per iteration, whether it happens or not, the constant also define the number of iterations of the algorithm which determines the unit of time).
- The rate of a first order reaction (such as decay or decreasing the counter of Δt_{repl}) is used "as such", whereas the rate of second order reaction (such as replication or diffusion of two individuals not in complex) is halved. The rate of third order reaction (e.g. complex diffusion) is divided by 6 and so on.

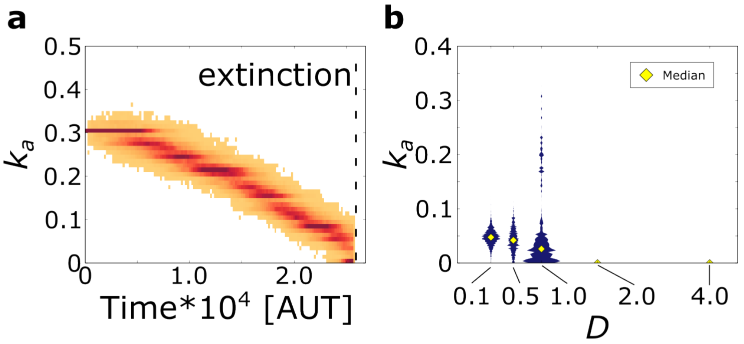

Figure S1: **a**: Evolutionary steady state of a replicators-only system with different diffusion coefficients D. Lattice size 512^2. **b**: In a mixed system extinction due to selection for becoming a template cannot be prevented. $D = 479.04$, meaning that out of 100 events, 96 on average are diffusion, each happening with probability $p_D = 0.998$. Lattice size 128^2. All other parameters are the same as in the main text.

S2 The effect of mixing and large diffusion rates

Mixing and large diffusion lead to extinction in the system with only replicators

Well-mixed system Mixing is approximated by greatly increasing the rate of diffusion. Unsurprisingly, selection for becoming a better template dominates when spatial patterns cannot form and global extinction happens when k_a reaches values too low to sustain replication (Fig. S1a).

Large diffusion rates In the main text, we have shown that low diffusion allows the persistence of replicators at the minimum viable association rate. This happens because local accumulation of replicators with too low values of k_a leads to their extinction, followed by re-invasion from neighbouring replicators. Increasing diffusion favours replicators with low k_a because it prevents their local accumulation, and allows them to exploit replicators further away from them, which leads to a lower steady state distribution of k_a (Fig. S1b). When diffusion is too large the system approaches a well-mixed conditions, k_a becomes too small to sustain replication, and extinction ensues. Interestingly, the limit value for diffusion rate before extinction ensues ($D = 1$) also displays the most variability. This is presumably due to the fact that local extinction of replicators happens on a somewhat larger spatial scale, so that the subsequent re-invasion endures for long enough to trigger some selection for larger k_a (see main text).

A moderate increase in diffusion rate does not qualitatively change results in replicators-parasites system

Increasing diffusion rate 5 folds does not lead to qualitatively different results (Fig. S2). We did not test the full range of β values due to computational load. However, replicators behave much like the default case ($D = 0.1$) in that 1) they reach the minimal viable k_a when no parasites are introduced, 2) they reach an evolutionary stable steady state for weaker parasites ($\beta = 1.30$), and maximise k_a when parasites are stronger ($\beta = 1.80$). Notice that, in general, an increase in diffusion makes spatial patterns larger. In order to contain such patterns for e.g. $D = 1$, the lattice should be much larger than what is computationally feasible.

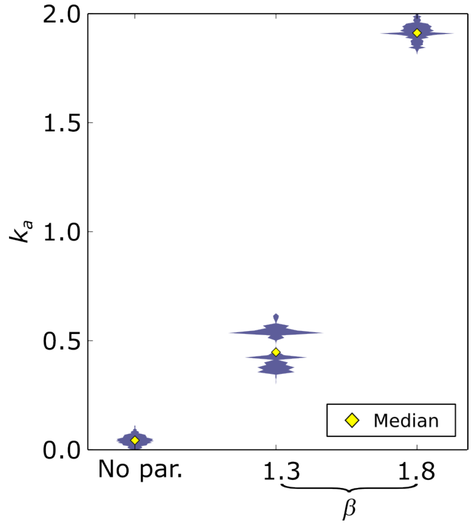

Figure S2: No qualitative difference observed in evolutionary steady state with a larger rate of diffusion. $D = 0.5$. Other parameters as in main text.

S3 Too weak parasites are not maintained in the system

When $\beta \leq 1$, parasites cannot prevent replicators from reaching low values of k_a and go extinct, as shown in Fig. S3.

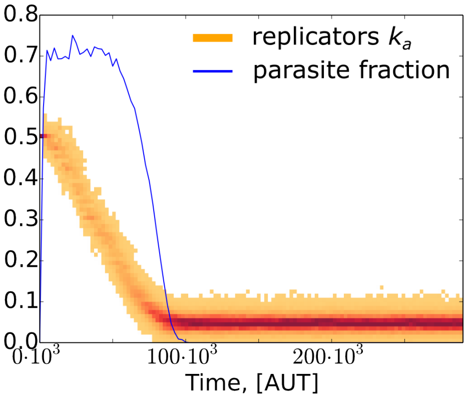

Figure S3: Evolutionary dynamics of replicators in the presence of very weak parasites, parasites go extinct when k_a becomes too small. $\beta = 1.0$, other parameters as in main text.

S4 Chaotic waves competition and selection for lower k_a

In order to illustrate how wave-level selection causes the decrease of k_a in replicators, we modified the set-up of the simulation by shaping the field as a long narrow strip. We initialised replicators and parasites in two waves, one at each end of the strip. In one wave replicators have larger k_a than in the other. We set mutation rates to zero to focus only on the selection process (the ecological competition). We let the two waves collide, as shown in Fig. S4. Although replicators with smaller k_a expand slower, they escape from the back of the original wave more frequently. The space behind the wave with weaker replicators is thus much fuller and works as a reservoir of replicators when the two initial waves collide and annihilate each other. The remaining empty space is then invaded by waves in which replicators have lower k_a.

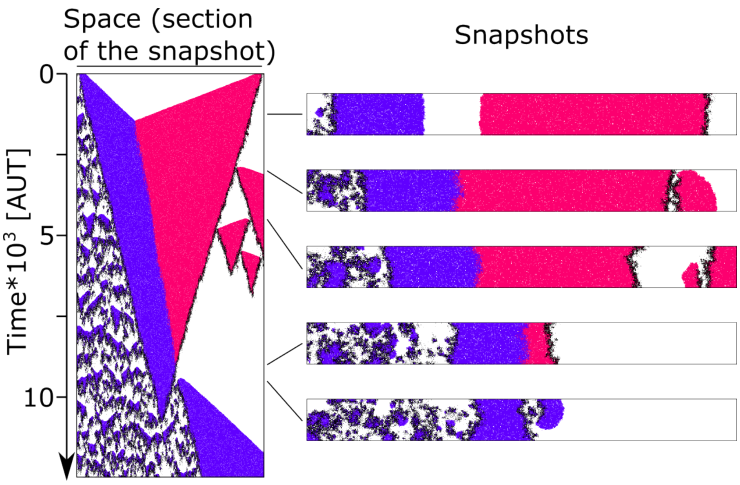

Figure S4: Wave level selection for lower k_a. Left: space-time plot of a replicator-parasite system (i.e. a stack of the middle row of the lattice at successive time points). Right: snapshots of the lattice at selected time points. Parasites (black) $\beta = 1.3$, replicators (indigo on the left) $k_a = 0.20$ and (magenta on the right) $k_a = 0.80$.

S5 Global extinction due to small lattice size

The size of spatial patterns can become comparable to that of the lattice when β and k_a are large. In this condition, the coexistence of multiple spatial patterns becomes impossible, and extinction can happen, as shown in Fig. S5. Larger lattices are therefore required to allow the evolutionary dynamics unfold properly (at the expenses of a larger computational load).

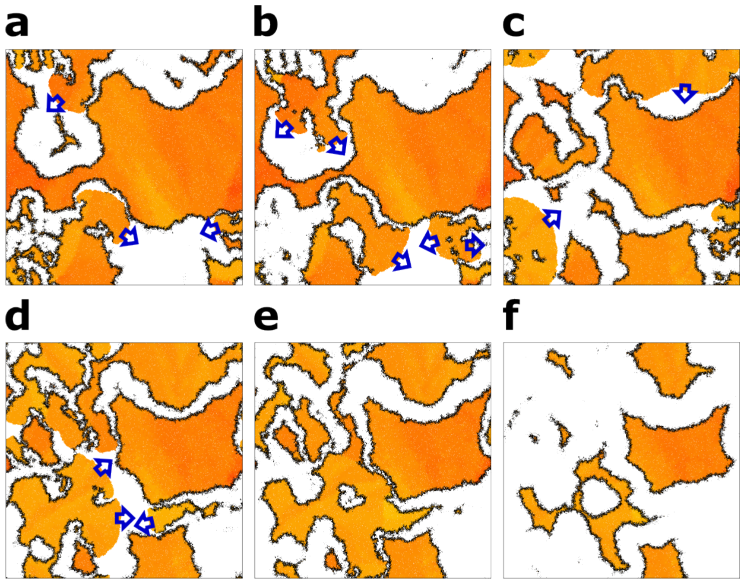

Figure S5: Small lattices lead to extinction when travelling waves become larger. **a**, **b**, **c**, **d**, **e**, **f**: Subsequent snapshots of the lattice. The expansion front of replicators is indicated by blue arrows. Parasites advantage $\beta = 1.7$. Notice that boundaries are wrapped.

S6 Stable waves competition and selection for larger k_a

When parasites are strong $\beta \leq 1.70$, travelling waves become larger and more stable, because limited (or no) escape is possible from their back. This means that, given the same parasite (i.e. the same $beta$) competition is determined by invasion into empty space, as shown in Fig. S6 (mutation rate is set to zero). Replicators with larger k_a are selected because they invade faster, and take over the expansion front.

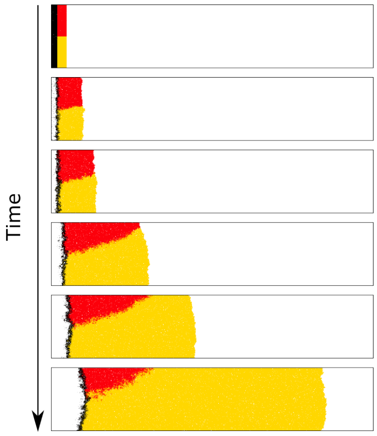

Figure S6: Replicators with larger values of k_a are selected in the presence of stronger parasites. Snapshots are at subsequent time steps. Parasites (black) $\beta = 1.80$, replicators above (red) $k_a = 1.0$, below (yellow) $k_a = 1.2$.

S7 Method for calculating the index of continuous empty space (Main Text Fig. 4)

The index in Main Text Fig. 4 is calculated as follows. For every combination of β and k_a shown in the figure, an ecological simulation (i.e. $\mu = 0$) is run with replicators and parasites. First a transient (5000 AUT) is let pass where the system self-organises and forms spatial patterns, then, 3x3 equally distant nodes (to minimise data correlations) in the field are screened for presence/absence of individuals. Every consecutive time step a node is empty, a counter is incremented. If the node becomes occupied, the number of consecutive time steps it spent as empty is recorded and the counter is re-set to zero. When the node turns empty again, the process can restart.

All the values of the counters are accumulated and a histogram is generated which represents the distribution of time spans (t_{empty}) nodes spent as empty. We used this as a proxy for the amount of continuous empty space a wave experiences. The log-transformed data can be well fitted by a linear function of the form $\alpha - \gamma t_{empty}$ (meaning that the original distribution is exponential), as shown in Fig. S7. $-\gamma$ is the index of continuous empty space of Main Text Fig. 4.

We chose $-\gamma$ because it increases when spatial patters generate larger amounts of empty space behind them.

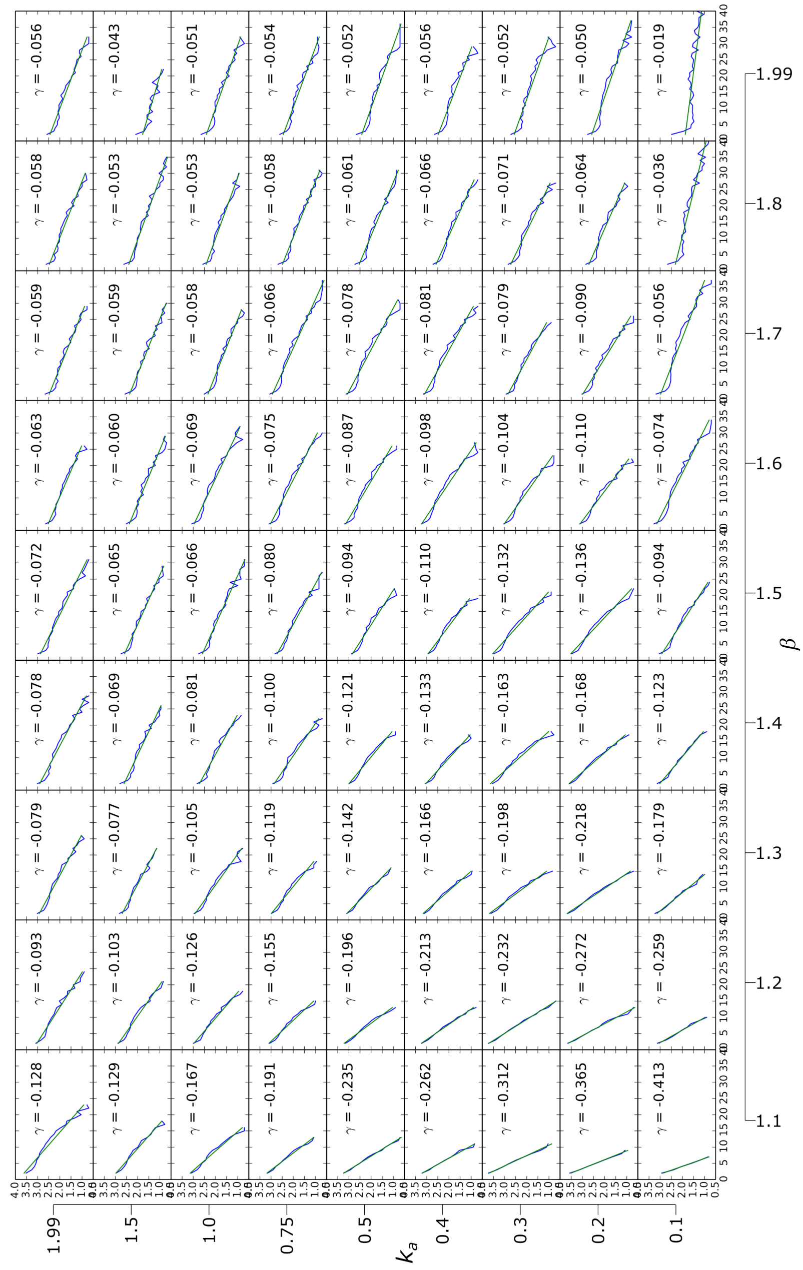

Figure S7: Fitting the time spans nodes spend as empty space with exponential distributions. Each plot is a combination of k_a and β ($\mu = 0$). Distribution is log-transformed (base 10), units on x-axis is AUT*500. Blue: original data, green: line-fit.

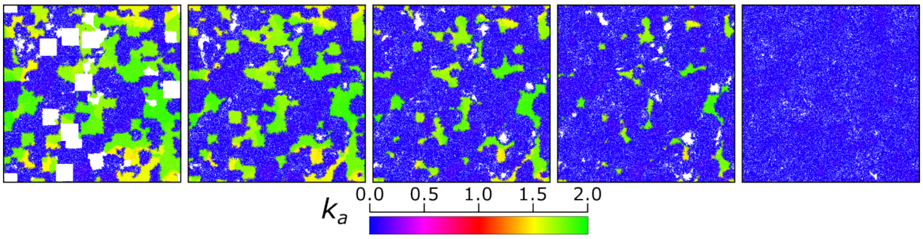

Figure S8: Interrupting ablations lead to the extinction of the species with higher k_a (green). Lattice size 1024^2.

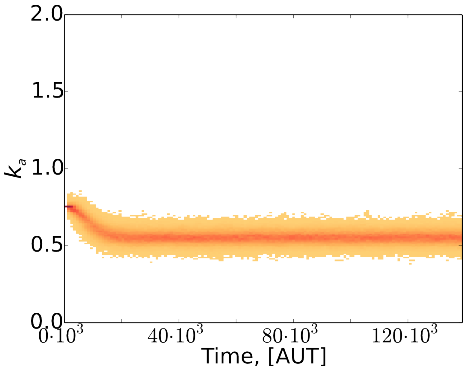

Figure S9: Increasing death rate does not lead to speciation. $k_{death} = 0.2$, other parameters as in main text.

S8 Ablations

Methods Periodical ablations are introduced in the system by deleting square patches every 50 AUT. In order to introduce n ablations of size η^2, n random coordinates are generated, which represent the centres of the ablations. The surface of size η^2 is then turned to empty, and the dynamics proceeds by normally (if an individual is in complex with one that is ablated, the complex breaks).

Ablations sustain the two species system Ablations of an intermediate size lead to the establishment of two distinct lineage in a system with only replicators. If ablations are stopped, however, the selection for increasing k_a disappears, and the species with the lowest k_a outcompetes the other, as shown in Fig. S8

Higher death rate does not lead to speciation In order to generate enough empty space to trigger selection for higher k_a, ablations must be of a minimum size. Point-sized ablations, for instance, are too small to achieve this. We modelled point size ablations by increasing the decay rate of replicators from the default $k_{death} = 0.03$ to 0.2 (i.e. 6.6 folds).

The evolutionary steady state value of k_a in Fig. S9 is larger than in Main Text Fig. 1a because individuals die much more frequently. However, we do not observe qualitative differences in evolutionary steady state behaviour, i.e. no speciation occurs.

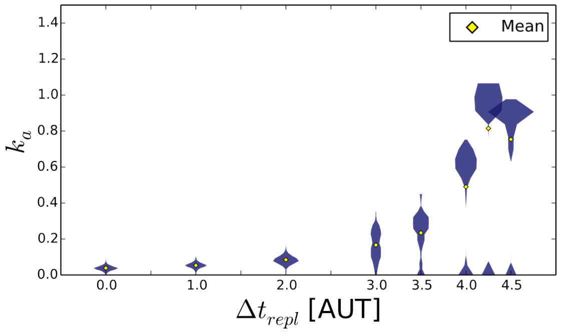

Figure S10: Evolutionary steady state values of k_a for replicators-only systems with longer replication times. $kdiss = 0.1$, other parameters as in main text.

S9 Longer replication time leads to speciation

The evolutionary separation of two lineages (one of which behaves as parasite) is observed for sufficiently duration of replication ($\Delta t_{repl} \leq 3.5$ AUT), as shown in Fig. S10. For

S10 Evolutionary transients during wave phase transition

When both β and k_a can mutate, we observe that evolution maximises both of them for $\Delta t_{repl} = 0$. Starting from smaller values of β and k_a, the system dynamically undergoes the phase transition between chaotic and stable waves. As lattice size must be large enough to contain the stable waves, spatial heterogeneities may lead to undergoing such phase transition locally, so that stable waves form in one part of the lattice (k_a and β increase) but not in another. Stable waves, however, are outcompeted by chaotic waves, because replicators in the latter can behave as parasites of the replicators in the former. Therefore, several "evolutionary attempts" may occur for a successful phase transition to actually happen at $\Delta t_{repl} = 0$,as shown in Fig. S11.

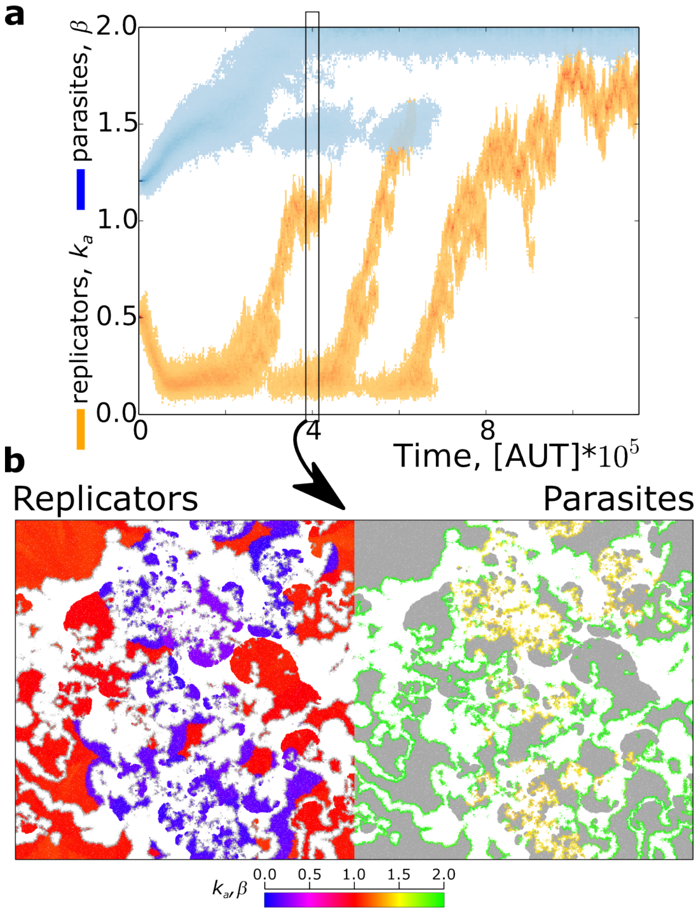

Figure S11: Repeated "evolutionary attempts" may be necessary to undergo the wave phase transition due to wave-level competition. **a**:Co-evolutionary dynamics of k_a and β for $\Delta t_{repl} = 0$. **b**: spatial distribution of replicators and parasites.

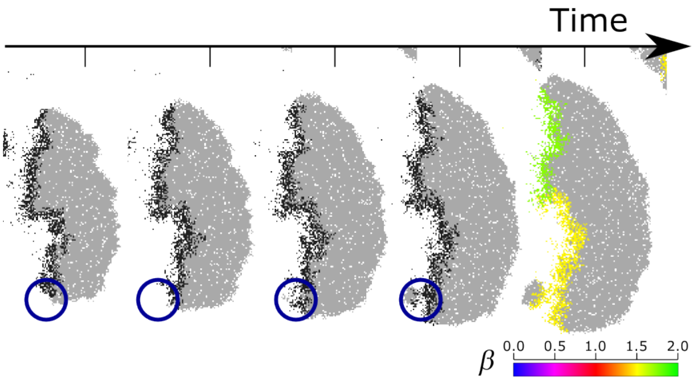

Figure S12: New waves are born from the side of older ones, when parasites are weaker. The same portion of the lattice is displayed at subsequent time steps (each 50 AUT). The circles highlight where the new wave is formed relative to the older one (all circles are at the same coordinates). Parameters as in main text.

S11 Limit behaviour for the generation of new waves

When β is very large, the longer-term stability of waves is threatened by the parasitic erosion of the invasion front. However, as the invasion front becomes narrower, small groups of replicators can be isolated from the side of the older wave, and establish a new one. Fig. S12 shows that this process selects for lower parasite strength because more waves are born from the sides of older waves when parasites are weaker, and constitutes the limit behaviour of the selection pressure that leads to chaotic travelling waves.

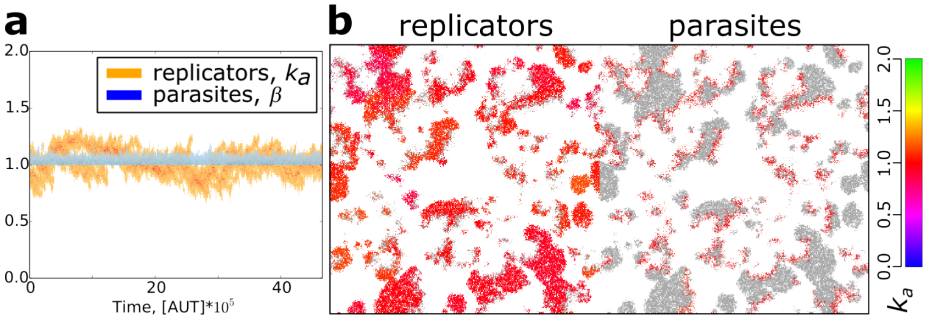

Figure S13: Spatial patterns allows indefinite persistence of replicators and parasites for $\Delta t_{repl} = 4.5$ when β cannot evolve below 1. Other parameters as in main text.

S12 Indefinite persistence for $\Delta t_{repl} = 4.5$ if $\beta > 1$

When $\Delta t_{repl} = 4.5$, parasites may drive themselves to extinction because they are selected to become worse templates than replicators (β evolves to lower than 1, see main text). The consequent loss of spatial patterns may lead to the extinction of replicators as well. Instead, both replicators and parasites persist indefinitely if parasites are not allowed to decrease β below 1, because travelling wave dynamics sustain the two species, as shown in Fig. S13.

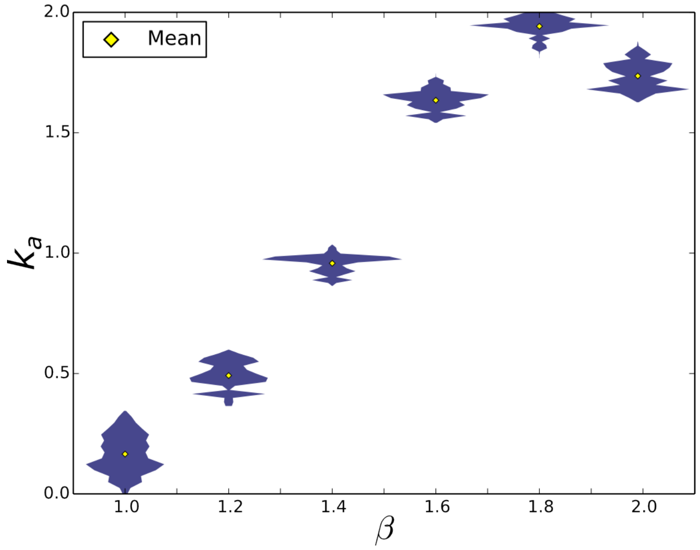

Figure S14: When only replicators mutate with longer replication times ($\Delta t_{repl} = 3$), they evolve to larger k_a in response to larger parasite advantage β.

S13 The effect of longer replication time is lost when only replicators evolve

Fig. S14 shows that when parasites are not allowed to co-evolve with replicators under conditions of longer replication time ($\Delta t_{repl} = 3$), we obtain qualitatively the same results of Main Text Fig. 2, i.e. that evolutionary steady state k_a increases with parasite strength. Notice that, even though we did not extensively test for the presence of a phase transition, the evolutionary maximisation of k_a is not observed in the co-evolutionary model.

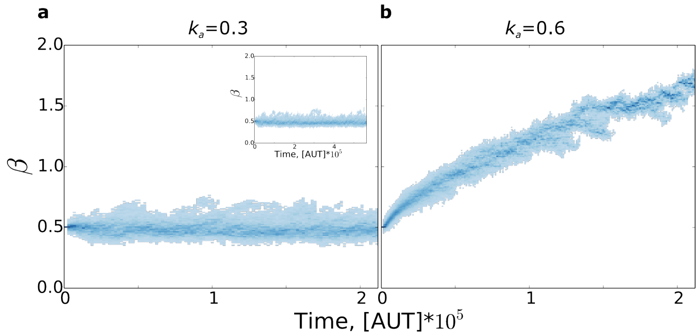

Figure S15: Parasite evolve to two different evolutionary steady state when replicators are weaker or stronger, and boundary conditions are fixed. **a**: Evolution of β while replicators have constant $k_a = 0.3$, inset: long term evolution; **b**: same, but $k_a = 0.6$. Other parameters as in main text.

S14 The evolution of parasites and previous results

In earlier work, it has been shown that parasites can evolve to lower probabilities of complex formation with a model similar to ours Takeuchi and Hogeweg (2009). In the main text we have seen that replicators can evolve to both higher or lower probabilities of complex formation (k_a) depending on parasite strength. Moreover, we have shown that replicator-parasites co-evolution can evolve in two different ways. Here we re-examine the results of Takeuchi and Hogeweg (2009) in the light of our results, and show that parasites also evolve to higher or lower complex formation rates as response to, respectively, weaker or lower replicators.

We set k_a constant and let β evolve. First we present results for $\Delta t_{repl} = 0$ and later we show how this compares to the case of $\Delta t_{repl} > 0$.

The evolution of β depends on k_a and on spatial pattern formation Fig. S15 shows that an evolutionary stable steady state exists for β when replicators' k_a is set at lower values (for $\Delta t_{repl} = 0$). This is the same result as in Takeuchi and Hogeweg (2009). However, we find that parasites evolve to increase β when association rate of replicators is larger.

Importantly, to recover the results from Takeuchi and Hogeweg (2009), we had to set the lattice boundaries to fixed (i.e. individuals disappear when they cross the edge of the lattice). In fact, stronger parasites organise in larger waves, which need more space to unfold properly. Therefore, fixed boundary conditions may confound results because larger waves are more likely to "fall out" of a lattice than smaller ones.

Because implementing larger lattices is computationally prohibitive, we turned boundary conditions to wrapped, so that larger waves did not disappear when they reached the boundaries, but instead re-entered from the opposite side of the lattice. We find that the parasites speciate and organise in both chaotic and stable travelling waves. While stronger parasites are more efficient at invading replicators (with which they form larger patterns), new waves are created from older ones where parasites are weaker (Fig. S16a). Thus, we observe co-existence Fig. S16b. Notice that the lineage with large β goes extinct because the associated waves become too large, and wrapped boundary conditions are no longer sufficient to counteract the effects of a small lattice. After extinction, evolution of a lineage which increases β occurs again.

Parasite evolution for longer replication time span For the sake of completeness, we repeated the analysis above for larger values of Δt_r (we tested $\Delta t_r = 1.5$ and 2.5). Results are in general agreement with above, i.e. parasites evolution leads to larger β when replicators' k_a is larger (Fig. S17). There are three points to make. First, before the phase transition, increasing k_a leads to a lower steady state value of β, in contrast to the case where replicators evolve with

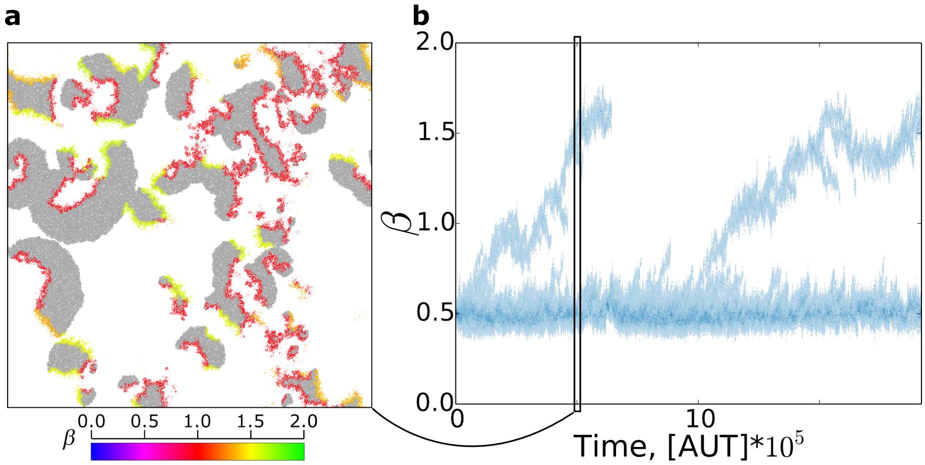

Figure S16: Co-existence of two lineages of parasites, mediated by the dynamics of different spatial patterns, for wrapped boundary conditions. **a**: A snapshot of the lattice. **b**: Evolution of β while replicators have constant $k_a = 0.3$.Other parameters as in main text.

fixed parasites (compare with Main Text Fig. 2 and Supplementary Fig. S14). Second, the phase transition occurs to larger value for larger value of k_a when Δt_{repl} is increased. Third, we observe long term co-existence of two parasite species for longer replication time span and large k_a (Fig. S17b, $k_a = 1.99$).

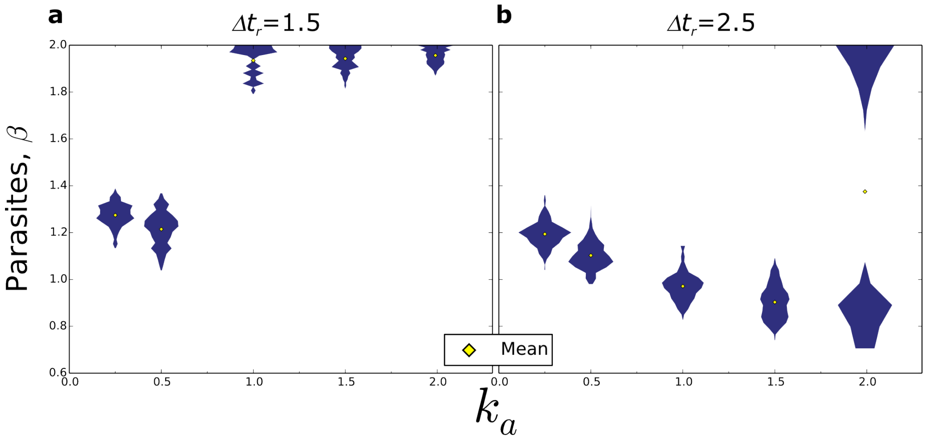

Figure S17: Parasite reach alternative evolutionary steady states depending on replicators' strength also larger replication time spans. **a**: $\Delta t_{repl} = 1.5$. **b**: $\Delta t_{repl} = 2.5$. Other parameters as in main text

References

de Boer, R. J. and Staritsky, A. D. Cash, 2000. http://bioinformatics.bio.uu.nl/rdb/software.html.

Takeuchi, N. and Hogeweg, P. Multilevel selection in models of prebiotic evolution ii: a direct comparison of compartmentalization and spatial self-organization. *PLoS computational biology*, 5(10):e1000542, 2009.
